# A deep error correction network for compressed sensing MRI

**DOI:** 10.1186/s42490-020-0037-5

**Published:** 2020-02-27

**Authors:** Liyan Sun, Yawen Wu, Zhiwen Fan, Xinghao Ding, Yue Huang, John Paisley

**Affiliations:** 1 Fujian Key Laboratory of Sensing and Computing for Smart City, Xiamen University, Xiamen, China; 2 Department of Electrical Engineering, Columbia University, New York, USA

**Keywords:** Fast imaging, Magnetic resonance imaging, Deep convolutional neural network

## Abstract

**Background:**

CS-MRI (compressed sensing for magnetic resonance imaging) exploits image sparsity properties to reconstruct MRI from very few Fourier k-space measurements. Due to imperfect modelings in the inverse imaging, state-of-the-art CS-MRI methods tend to leave structural reconstruction errors. Compensating such errors in the reconstruction could help further improve the reconstruction quality.

**Results:**

In this work, we propose a DECN (deep error correction network) for CS-MRI. The DECN model consists of three parts, which we refer to as modules: a guide, or template, module, an error correction module, and a data fidelity module. Existing CS-MRI algorithms can serve as the template module for guiding the reconstruction. Using this template as a guide, the error correction module learns a CNN (convolutional neural network) to map the k-space data in a way that adjusts for the reconstruction error of the template image. We propose a deep error correction network. Our experimental results show the proposed DECN CS-MRI reconstruction framework can considerably improve upon existing inversion algorithms by supplementing with an error-correcting CNN.

**Conclusions:**

In the proposed a deep error correction framework, any off-the-shelf CS-MRI algorithm can be used as template generation. Then a deep neural network is used to compensate reconstruction errors. The promising experimental results validate the effectiveness and utility of the proposed framework.

## Background

MRI (Magnetic resonance imaging) is an important medical imaging technique with high resolution in soft tissues, low radiations, but the slow imaging speed is a major drawback of MRI. CS (Compressed sensing) theory [1, 2] has been a significant development of the signal acquisition and reconstruction process that has allowed for significant acceleration of MRI with less k-space measurements. The CS-MRI problem can be formulated as the optimization

1 $$ \hat x = \mathop {\arg \min }\limits_{x} \left\| {{F_{u}}x - y} \right\|_{2}^{2} + \sum\limits_{i} {{\alpha_{i}}{\Psi_{i}}\left(x \right)},  $$

where *x*∈*C*^*N*×1^ is the complex-valued MRI to be reconstructed, *F*_*u*_∈*C*^*M*×*N*^ is the under-sampled Fourier matrix and *y*∈*C*^*M*×1^ (*M*≪*N*) are the k-space data measured by the MRI machine. The first data fidelity term ensures agreement between the Fourier coefficients of the reconstructed image and the measured data, while the second term regularizes the reconstruction to encourage certain image properties such as sparsity in a transform domain.

Recently, the compressed sensing MRI is approved by the FDA (Food and Drug Administration) to two main MRI vendors: GE and Siemens [3]. As the growing needs for application of compressed sensing MRI, improving reconstruction accuracy of the CS-MRI is of great significance. In this paper, we propose a deep learning framework called DECN (deep error correction network) in which an arbitrary CS-MRI inversion algorithm is combined with a deep learning error correction network. The network is trained for a specific inversion algorithm to exploit structural consistencies in the errors they produce. The final reconstruction is found by combining the information from the original algorithm with the error correction of the network.

A lot of previous works focus on proposing appropriate regularizations that lead to better MRI reconstructions. In the pioneering work of CS-MRI called SparseMRI [4], this regularization adds an *ℓ*_1_ penalty on the wavelet coefficients and the total variation of the reconstructed image. Based on SparseMRI, more efficient optimization methods have been proposed to optimize this objective, such as TVCMRI (Total Variation *ℓ*_1_ Compressed MR Imaging) [5], RecPF (Reconstruction From Partial Fourier Data) [6] and FCSA (Fast Composite Splitting Algorithm) [7]. Variations on the wavelet penalty exploit geometric information of MRI, such as PBDW/PBDWS (Patch Based Directional Wavelet) [8, 9] and GBRWT (Graph Based Redundant Wavelet Transform) [10], for improved results. Dictionary learning methods [11–14] have also been applied to CS-MRI reconstruction, as have nonlocal priors such as NLR (Non-Local Regularization) [15], PANO (Patch Based Non-Local Operator) [16] and BM3D-MRI (Block-Matching 3D MRI) [17]. These previous works can be considered sparsity-promoting regularized CS-MRI methods that are optimized using iterative algorithms. They also represent images using simple single layer features that are either predefined (e.g., wavelets) or learned from the data (e.g., dictionary learning).

Recently, deep learning approaches have been introduced for the CS-MRI problem, achieving state-of-the-art performance compared with conventional methods. For example, an end-to-end mapping from input zero-filled MRI to a fully-sampled MRI was trained using the classic CNN model in [18], or its residual network variant in [19]. In the residual network proposed in [19], a global shortcut is applied to enforce a U-Net architecture input with a zero-filled MRI to learn the difference between the full-sampled MRI and its zero-filled one.

Although the work [19] shares the idea of residual learning with our approaches, there are some major differences between the two methods. In our model, the network design is motivated by exploiting the structural residual errors left by general reconstruction algorithms, the error correction module input with both the zero-filled MRI and guide image to learn the residual between the full-sampled MRI and guide image. If the error correction module is an identical mapping, the proposed DECN will be turned into the similar architecture to the compared model. However, our deep error correction network can be seen as a generalization of the compared network since the error correction module could be any off-the-shelf CS-MRI algorithms. Better reconstruction a guide module achieves, the smaller residual errors and the improvement under our framework are. Besides, for the input of the error correction module, the concatenation design of the zero-filled and guide MRI is motivated and justified by the observation that guide image produced by an off-the-shelf MRI reconstruction algorithm is imperfect and lose details compared with zero-filled MRI, which is not discussed in the compared model.

Greater integration of the data fidelity term into the network has resulted in a DC-CNN (Deep Cascade CNN) [20, 21]. The conventional iterative optimization is also unfolded as deep neural networks [22] called ADMM-Net where the transform domain is learnable in a full supervised manner. The adversarial training strategy is also introduced in CS-MRI [23–25] to help the reconstructed MRI more realistic. In DAGAN proposed in [23], frequency domain information is incorporated in the adversarial learning framework. A refinement U-net is designed as generator with a content loss to preserve details. A cyclic loss is introduced with a chain refinement strategy is proposed in [24] called RefineGAN for compressed sensing MRI. Similar GAN architecture is also evaluated in rapid MRI in [25].

Compared with previous models proposed for CS-MRI inversion, deep learning is able to capture more intricate patterns within the data in both image domain and frequency domain [26, 27], which leads to their improved performance.

Previous work has also tried to exploit regularities in the reconstruction error in different ways. In the popular dynamic MRI reconstruction method k-t FOCUSS (k-t FOCal Underdetermined System Solver) [28, 29], the original signal is decomposed into a predicted signal and a residual signal. The predicted signal is estimated by temporal averaging, while the highly sparse residual signal has a *l*_1_-norm regularization. An iterative feature refinement strategy called IFR-CS for CS-MRI was proposed in [30]. The IFR-CS method is an iterative optimization based approach. In certain iteration in this model, a sparsity promotion module using total variation (TV) is applied on the input noisy MR image to obtain a rough estimation first. Then a manually designed feature extractor is used on the rough estimation to generate a feature. The feature is calibrated by the difference between the noisy MR image input into the sparsity promotion module and the rough estimation to produce a refined feature. Then this refined feature is added back to the rough estimation to obtain the output in the iteration. The optimization iterates till it converges. Compared with our deep error correction network (DECN), the feature extraction of IFR-CS is hand-crafted, whereas a deep network can better extract features automatically in DECN. Also DECN model is more general because all compressed sensing MRI methods can be used to generate guide/rough image. The IFR-Net is a variant of the IFR-CS method using deep convolutional neural networks [31]. The IFR-Net unrolls the IFR-CS using deep learning architecture, which improves the transform domain and feature learning. The IFR-Net shares similarities in using deep models for error correction with proposed DECN model although it is based on IFR-CS formulation. In [32], the k-space measurements are divided into high and low frequency regions and reconstructed separately. In [33] the MR image is decomposed into a smooth layer and a detail layer which are estimated using total variation and wavelet regularization separately. In [34], the low frequency information is estimated using parallel imaging techniques. These methods each employ a fixed transform basis.

## Methods

### Problem formulation

Exploiting structural regularities in the reconstruction error of CS-MRI is a good approach to compensate for imperfect modeling. Starting with the standard formulation of CS-MRI in Eq. 1, we formulate our objective function as

2 $$ \hat x = \mathop {\arg \min }\limits_{x} \left\| {{F_{u}}x - y} \right\|_{2}^{2} + \alpha \left\| {x - {x_{p}}} \right\|_{2}^{2},  $$

where *x*_*p*_ is an intermediate reconstruction of the MRI. Due to the imperfect modeling, we model this intermediate reconstruction *x*_*p*_ as the summation of a “guidance” image $\overline x_{p}$ and the error image of the reconstruction *Δx*_*p*_,

3 $$ x_{p} = {\overline x_{p}} + \Delta x_{p}.  $$

Substituting this into Eq. 2, we obtain

4 $$ \hat x = \mathop {\arg \min }\limits_{x} \left\| {{F_{u}}x - y} \right\|_{2}^{2} + \alpha \left\| {x - \left({\overline x_{p} + \Delta x_{p}} \right)} \right\|_{2}^{2}.  $$

The guidance image $\overline x_{p}$ is the reconstructed MRI using any chosen CS-MRI method; thus *x*_*p*_ can be formed using existing software prior to using our proposed method for the final reconstruction. The reconstruction error *Δx*_*p*_ is between the ground truth full-sampled MRI *x*_*fs*_ and the reconstruction $\overline x_{p}$. Since we don’t know this at testing time, we use training data to model this error image with a neural network ${f_{\theta }(\mathcal {X}) }$, where *θ* represents the network parameters and $\mathcal {X}$ is the input to the network. Thus, Eq. 4 can be rewritten as

5 $$ \hat x = \mathop {\arg \min }\limits_{x,\theta} \left\| {{F_{u}}x - y} \right\|_{2}^{2} + \alpha \left\| {x - \overline x_{p} - {f_{\theta}(\mathcal{X}) }} \right\|_{2}^{2}.  $$

For a new MRI, after obtaining the guidance image $\overline x_{p}$ (using a pre-existing algorithm) and the well-learned mapping $\Delta x_{p} = {f_{\theta }(\mathcal {X}) }$ (using a feed-forward neural network trained on data), the proposed framework produces the final output MRI by solving the least square problem of Eq. 5.

### Deep error correction network (DECN)

**Fig. 1:**
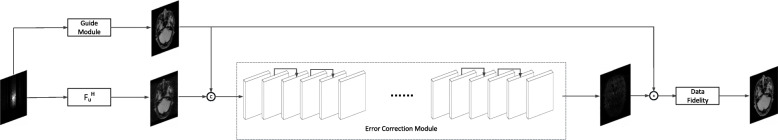
The proposed Deep Error Correction Network (DECN) architecture consists of three modules: a guide module, an error correction module, and a data fidelity module. The input of the error correction module is the concatenation of the zero-filled compressed MR samples and guidance image while the corresponding training label is the reconstruction error △*x*_*p*_. After the error correction module is trained, the guidance image and feed-forward approximation of the reconstruction error for a test image are used to produce the final reconstructed MRI

Following the formulation of our CS-MRI framework above and in Fig. 1, we turn to a more detailed discussion of the optimization procedure. We next discuss each module of the proposed Deep Error Correction Network (DECN) framework.

#### Guide module

With the guide module, we seek a reconstruction of the MRI $\overline x_{p}$ that approximates the fully-sampled MRI using a standard “off-the-shelf” CS-MRI approach. We denote this as

6 $$ \overline x_{p} = {\text{invMRI}}\left(y \right).  $$

**Fig. 2:**
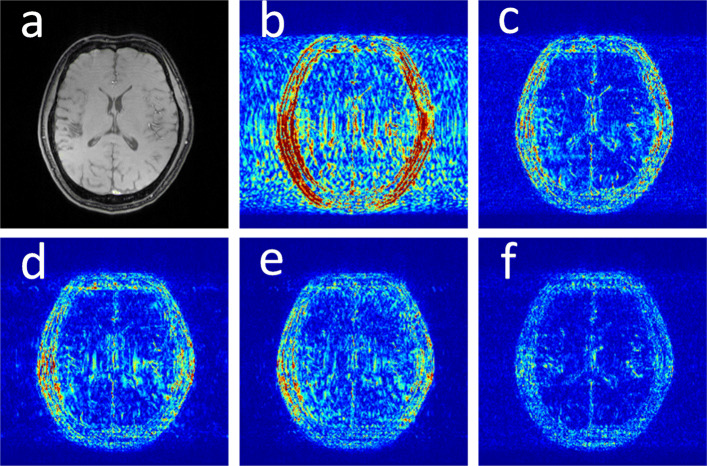
The reconstruction error of a brain MRI using zero-filled, TLMRI, PANO, GBRWT and DC-CNN under 1D 30% under-sampling mask. **a** Fully-sampled MRI, **b** Zero-filled error, **c** TLMRI error, **d** PANO error, **e** GBRWT error, **f** DC-CNN error

We first illustrate with reconstructions for three CS-MRI methods: TLMRI (transform learning MRI) [14], PANO (patch-based nonlocal operator) [16] and GBRWT (graph-based redundant wavelet transform) [10]. The PANO and GBRWT models achieve impressive reconstruction qualities because they use an nonlocal prior and adaptive graph-based wavelet transform to exploit image structures. In TLMRI, the sparsifying transform learning and the reconstruction are performed simultaneously in more efficient way than DLMRI (dictionary learning MRI) [11]. The three methods represent the state-of-the-art performance in the non-deep CS-MRI models. In Fig. 2, we show the reconstructions error for zero-filled (itself a potential reconstruction “algorithm”), TLMRI, PANO and GBRWT on a complexed-valued brain MRI using 30% Cartesian under-sampling. The error display ranges from 0 to 0.2 with normalized data. The parameter setting will be elaborated in the Results section. We observe the reconstruction errors show high degree of sparsity and obvious image structures. From sparse representation theory, a more sparse signal can be recovered with less measurements [1, 35], which provide a solid ground that the sparse structural reconstruction error can be well approximated.

We also consider the representative deep learning DC-CNN model [20] as the guide module. We also give the reconstruction error in Fig. 2. We observe the zero-filled, TLMRI, PANO, GBRWT and DC-CNN models all suffer the structural reconstruction errors, while the DC-CNN model achieves the highest reconstruction quality with minimal errors because of its powerful model capacity. Another advantage of this CNN model is that, once the network is trained, testing is very fast compared with conventional sparse-regularization CS-MRI models. This is because no iterative algorithm needs to be run for optimization during testing since the operations are a simple feed forward function of the input. We compare the reconstruction time of TLMRI, PANO, GBRWT and DC-CNN *for testing* for Fig. 2 in Table 1. Note the DC-CNN is implemented on GPU and other non-deep methods are implemented on CPU. However, the major reason for the difference in running speed among deep and non-deep models lies in the non-iterative forward reconstruction property of the deep models when testing.

**Table 1:** Reconstruction time of PANO, TLMRI, GBRWT and DC-CNN

	PANO	TLMRI	GBRWT	DC-CNN
Runtime (seconds)	11.37s	127.67s	100.60s	0.04s

#### Error correction module

Using the guidance image $\overline x_{p}$, we can train a deep error correction module on the residual. To perform this task, we need access during training to pairs of the true, fully sampled MRI *x*_*fs*_, as well as its reconstruction $\overline x_{p}$ found by manually undersampling the k-space of this image according to a pre-defined mask and inverting. We then optimize the following objective function over network parameter *θ*,

7 $$ \hat \theta = \mathop {\arg \min }\limits_{\theta} \frac{1}{2}\left\| {\left({{x_{fs}} - {{\bar x}_{p}}} \right) - {f_{\theta} }\left(\mathcal{Z}(y),\overline x_{p} \right)} \right\|_{2}^{2},  $$

where $\mathcal {Z}(y)$ indicates the reconstructed MRI using zero-filled and the input to the error correction module $\mathcal {X}$ is the concatenation of the zero-filled MRI $\mathcal {Z}(y)$ and the guidance MRI $\overline x_{p}$ as shown in Fig. 1. Therefore, the error-correcting network is learning how to map the concatenation of the zero-filled, compressively sensed MRI and the guidance image to the residual of the true MRI using a corresponding off-the-shelf CS-MRI inversion algorithm. Now we give the rationales and explanations for the concatenation operation.

In the CS-MRI inversions, the zero-filled MR images usually serve as the starting point in the iterative optimization. Although the iterative de-aliasing can effectively remove the artifacts and achieve much more pleasing visual quality compared with zero-filled reconstruction, the distortion and information loss is inevitable in the reconstruction. To further illustrate this phenomenon, we compare the pixel-wise reconstruction errors among the zero-filling reconstruction and other non-deep reconstruction models of the MR image in Fig. 2.

We take the difference between the absolute reconstruction error of the reconstructed MRI produced by compared CS-MRI methods and zero-filled and only keep the nonnegative values, which can be formulated as

8 $$ m_{d} = {\left({\left| {{x_{fs}} - {\overline x_{p}}} \right| - \left| {{x_{fs}} - {\mathcal{Z}}(y)} \right|} \right)_ + }.  $$

**Fig. 3:**
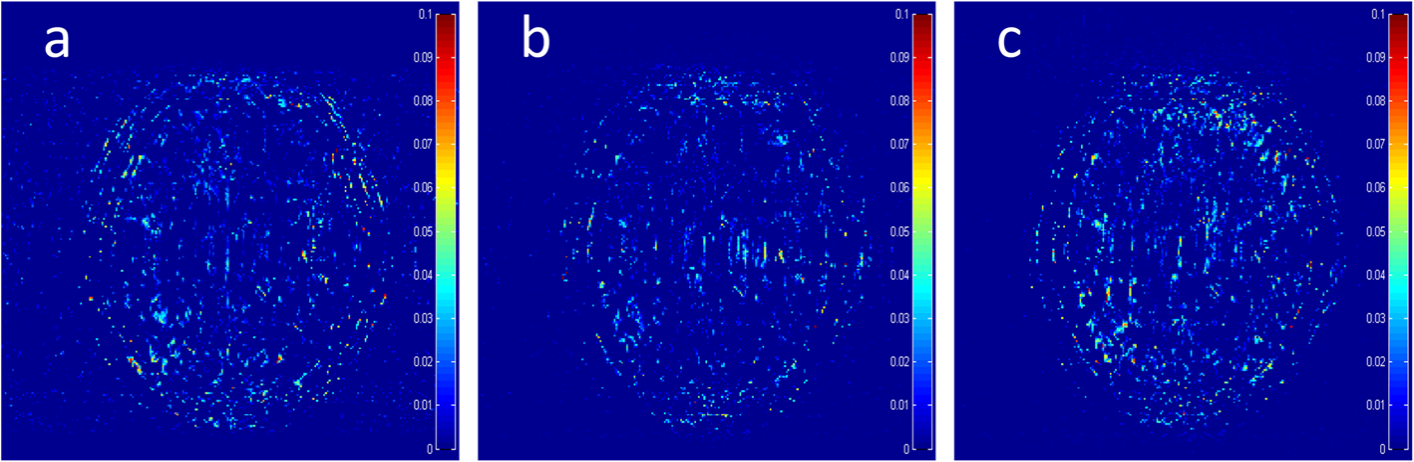
The filtered difference map md between the reconstruction errors of the zero-filled reconstruction and recent CS-MRI inversions. **a** TLMRI *m*_*d*_, **b** PANO *m*_*d*_, **c** GBRWT *m*_*d*_

Where the operator (·)_+_ set the negative values to zero. We only keep the nonnegative values in the map, which results the filtered difference map. We show the corresponding filtered difference map *m*_*d*_ in Fig. 3 in the form of color map ranging from 0 to 0.1 with a 1D Cartesian 30% undersamling mask. On certain pixel of the reconstruction, if the guide reconstruction is less accurate compared with zero-filling, the difference on this pixel would be positive. Because we hope to find out if the zero-filled MRI is more accurate on some pixels, the negative values are not our interests and filtered. In the filtered difference map, the bright region means the better accuracy of zero-filled reconstruction. We observe the zero-filling reconstruction provide better reconstruction accuracy compared with different methods on some regions, indicating the information loss in the reconstruction occurs.

To alleviate the information loss in the guide module, we introduce the concatenation operation to utilize the information from both the zero-filled MR image and guidance image as the input to the error correction network. In later Discussion section, we further validate it by the ablation study.

We again note that the network ${f_{\theta } }\left (\mathcal {Z}(y),\overline x_{p} \right)$ is paired with a particular inversion algorithm invMRI(*y*), since each algorithm may have unique and consistent characteristics in the errors they produce. The network ${f_{\theta } }\left (\mathcal {Z}(y),\overline x_{p} \right)$ can be any deep learning network trained using standard methods.

#### Data fidelity module

After the error correction network is trained, for a new undersampled k-space data *y* for which the true *x*_*fs*_ is unknown, we use its corresponding guidance image $\overline x_{p} = \text {invMRI}(y)$ and the approximated reconstructed error ${f_{\theta }}\left ({\mathcal {Z}(y),\overline x_{p}} \right)$ to optimize the data fidelity module by solving the following optimization problem

9 $$ {}\hat x = \mathop {\arg \min }\limits_{x} \left\| {{F_{u}}x - y} \right\|_{2}^{2} + \alpha \left\| {x - \left({{{\bar x}_{p}} + {f_{\theta }}\left({\mathcal{Z}(y)},\overline x_{p} \right)} \right)} \right\|_{2}^{2}.  $$

The data fidelity module is utilized in our proposed DECN framework to correct the reconstruction by enforcing greater agreement at the sampled k-space locations [11, 12]. Using the properties of the fast Fourier transform (FFT), we can simplify the optimization by working in the Fourier domain using the common technique described in, e.g., [12]. The optimal values for $\hat x$ in k-space can be found point-wise. This yields the closed-form solution

10 $$ \hat x = {F^{H}}\frac{{{FF}_{u}^{H}y + \alpha F\left({{{\overline x }_{p}} + {f_{\theta} }\left({\mathcal{Z}(y),\overline x_{p}} \right)} \right)}}{{{FF}_{u}^{H}{F_{u}}{F^{H}} + \alpha I}}.  $$

The regularization parameter *α* is usually set very small in the noise-free environment. We found that *α*=5*e*−5 worked well in our low-noise experiments.

## Results

### Data

In the experiment section, we present experimental results using complex-valued MRI datasets. The T1 weighted MRI dataset (size 256×256) is acquired on 40 volunteers with total 3800 MR images at Siemens 3.0T scanner with 12 coils using the FLASH (Fast Low Angle SHot) sequence (TR/TE = 55/3.6*ms*, 220 *mm*^2^ field of view, 1.5*mm* slice thickness). The SENSE (SENSitivity Encoding) reconstruction is introduced to compose the gold standard full k-space, which is used to emulate the single-channel MRI. For SENSE reconstruction, each coil receives partial MRI signal and produce the corresponding parallel MRI images. Then the coil sensitivity maps are computed for each coil and used for generating the full-sampled MRI data by matrix inversion. The similar simulation setting can be found in [8]. We randomly select 75% MR images as training set, 5% as validation set and 20% as testing set. Informed consent was obtained from the imaging subject in compliance with the Institutional Review Board policy. The magnitude of the full-sampled MR image is normalized to unity by dot dividing the image by its largest pixel magnitude. The real and imaginary parts of a complex MRI data are input into the deep neural networks in two-channel manner [20].

We also validate our deep error correction network on the publicized MRI brain datasets MRBrainS13 [36]. The dataset is acquired at UMC Utrecht from patients. Each imaging subject is scanned to acquire multimodality MRI brain data including T1, T1-IR and T2-FLAIR modalities. Here we use T2-FLAIR MRI throughout our paper. Bias correction has been applied on all scans and the data of each patient aligned. The voxel size is 0.958*mm*×0.958*mm*×3.00*mm*. There are total 5 scans in the training datasets. We use the fifth scan for testing and the rest 4 scans for training.

Under-sampled k-space measurements are manually obtained via Cartesian and Random sampling mask with random phase encodes. Different undersampling ratios are adopted in the experiments.

### Network architecture

For the deep guide module (i.e., learning $\overline x_{p}$), we use the CNN architecture called deep cascade CNN [20], where the non-adjustable data fidelity layer is also incorporated into the model. This guide module consists of four blocks. Each block is formed by four consecutive convolutional layers with a shortcut and a data fidelity layer. For each convolutional layer, except the last one within a block, there are total of 64 feature maps. We use ReLU (Rectified Linear Unit) [37] as the activation function.

For the error correction module (i.e., learning $f_{\theta }(\mathcal {Z}(y),\overline x_{p})$), we adopt the network architecture shown in Fig. 1. There are 18 convolutional layers with a skip layer connection as proposed in [38, 39] to alleviate the gradient vanish problem. We again adopt ReLU as the activation function, except for the last layer where the identity function is used to allow negative values. All convolution filters are set to 3×3 with stride set to 1.

### Experimental setup

We train and test the two deep algorithms using Tensorflow [40] for the Python environment on a NVIDIA GeForce GTX 1080 with 8GB GPU memory. Padding is applied to keep the size of features the same. We use the Xavier method [41] to initialize the network parameters, and we apply ADAM [42] with momentum. The implementation uses the initialized learning rate 0.0001, first-order momentum 0.9 and second momentum 0.99. The weight decay regularization parameter is set to 0.0005. The size of training batch is 4. We report our performance after 20000 training iteration of DC-CNN guide module and 40000 iterations of error correction module.

In the guidance module, we implement the state-of-the-art CS-MRI models with the following parameter settings. In TLMRI [14], we set the data fidelity parameter 1*e*6/(256×256), the patch size 36, the number of training signals 256×256, the sparsity fraction 4.6*%*, the weight on the negative log-determinat+Frobenius norm terms 0.2, the patch overlap stride 1, the DCT (Discrete Cosine Transform) matrix is used as initial transform operator, the iterations 50 times for optimization. The above parameter setting follows the advices from the author [14]. In PANO [16], we use the implementation with parallel computation provided by [16]. The data fidelity parameter is set 1*e*6 with zero-filled MR image as initial reference image. The non-local operation is implemented twice to yield the MRI reconstruction. In GBRWT [10], we set the data fidelity parameter 5×1*e*3. The Daubechies redundant wavelet sparsity is used as regularization to obtain the reference image. The graph is trained 2 times.

### Experimental results

**Fig. 4:**
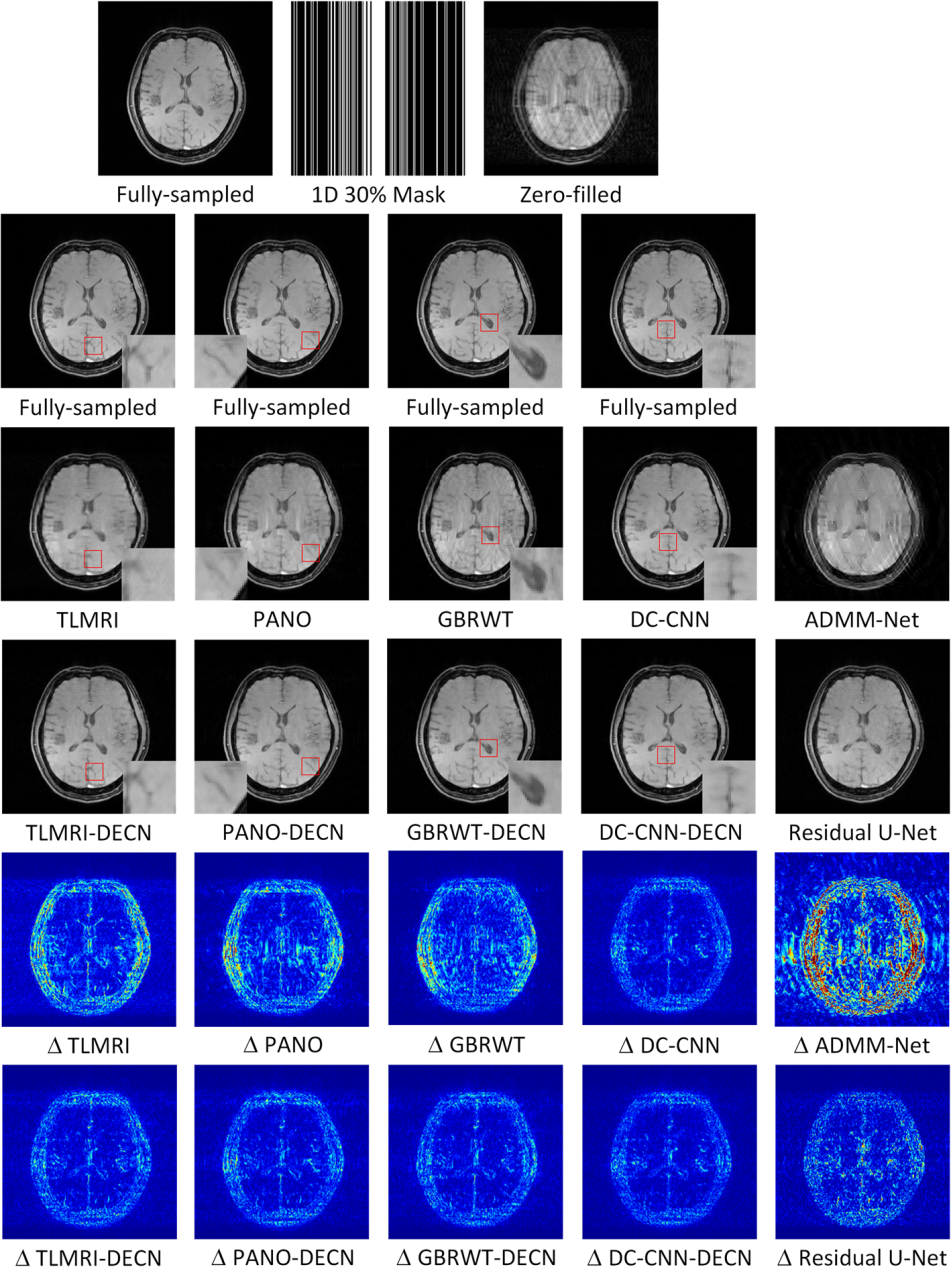
We show the reconstruction results of our DECN model with local area magnification. We also show the reconstruction error for our DECN model under different guide module in the last row

**Fig. 5:**
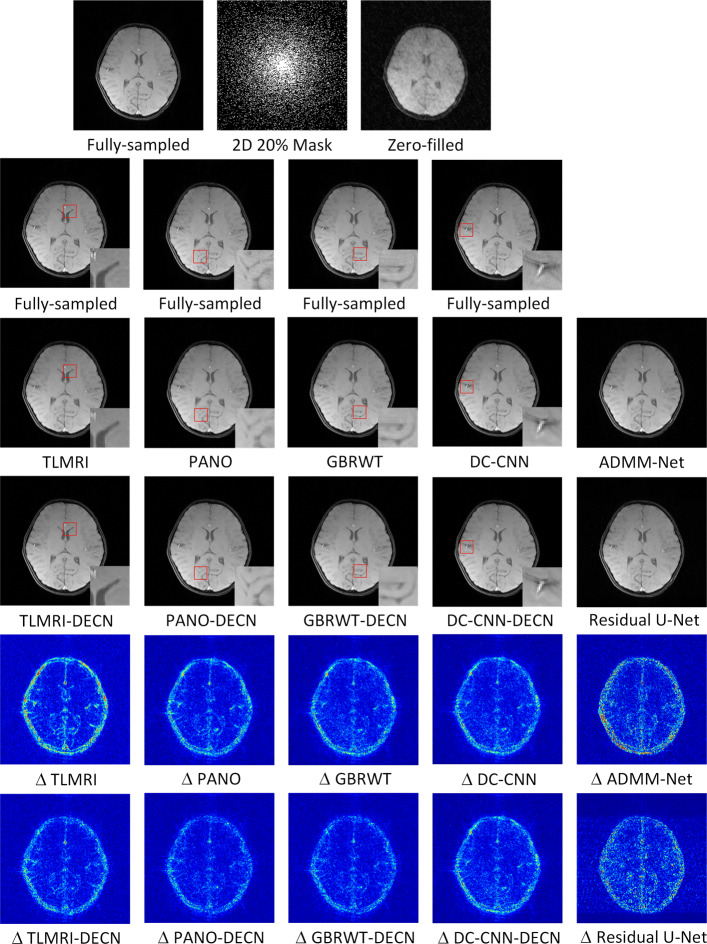
We show the reconstruction results of our DECN framework with local area magnification on Random 20% under-sampling mask. We also show the reconstruction error for our DECN model under different guide module in the last row

We evaluate the proposed DECN framework using PSNR and SSIM (structural similarity index) [43] as quantitative image quality assessment measures. We give the quantitative reconstruction results of all the test data on different under-sampling patterns and different under-sampling ratios in Table 2. We show the Cartesian 30% under-sampling mask in Fig. 4 and the Random 20% under-sampling mask in Fig. 5. We observe that DECN improved all off-the-shelf CS-MRI inversion methods on all the under-sampling patterns. Since the 2D Random mask enjoys the more incoherence than the 1D Cartesian mask with the same under-sampling ratio, the CS-MRI achieves better reconstruction quality on the Random masks. We observe all different regular CS-MRI inversions can be improved in PSNR and SSIM metrics. Also, we observe the plain DC-CNN model already achieves better reconstruction accuracy than other compared models, leaving less structural errors for the error correction module, leading to less performance improvement. However, in the field of medical imaging where the quantitative accuracy matters, the small improvement in reconstruction quality is also valuable.

**Table 2:** The objective evalution on the regular CS-MRI inversions and their DECN frameworks

Sampling pattern	Cartesian under-sampling						Random under-sampling					
Sampling ratio	20%		30%		40%		20%		30%		40%	
Evaluation index	PSNR	SSIM	PSNR	SSIM	PSNR	SSIM	PSNR	SSIM	PSNR	SSIM	PSNR	SSIM
TLMRI	31.27	0.864	32.86	0.868	35.99	0.896	35.13	0.878	36.46	0.882	37.26	0.891
PANO	30.71	0.858	32.65	0.889	37.40	0.940	36.94	0.931	39.36	0.949	40.74	0.957
GBRWT	30.61	0.853	32.27	0.879	37.19	0.932	36.81	0.908	39.16	0.932	40.72	0.944
DC-CNN	32.58	0.885	34.67	0.905	39.52	0.955	38.54	0.937	40.91	0.953	42.47	0.961
TLMRI-DECN	32.77	0.876	34.41	0.891	38.62	0.944	37.60	0.930	39.54	0.944	40.72	0.949
PANO-DECN	32.57	0.864	34.43	0.891	39.27	0.953	38.51	0.940	40.88	0.956	42.42	0.963
GBRWT-DECN	32.58	0.869	34.41	0.891	39.07	0.950	38.48	0.940	40.79	0.955	42.36	0.963
DC-CNN-DECN	33.06	0.898	35.34	0.922	39.92	0.956	38.86	0.939	41.06	0.954	42.58	0.962
△ TLMRI	1.50	0.012	1.55	0.023	2.63	0.048	2.47	0.052	3.08	0.062	3.46	0.068
△ PANO	1.86	0.006	1.78	0.002	1.87	0.012	1.57	0.010	1.52	0.006	1.68	0.006
△ GBRWT	1.97	0.016	2.14	0.012	1.88	0.018	1.67	0.032	1.63	0.023	1.64	0.019
△ DC-CNN	0.48	0.013	0.67	0.017	0.40	0.010	0.32	0.002	0.15	0.001	0.11	0.010

In Fig. 4, we show reconstruction results and the corresponding error images of an example from the test data on the 1D 30% under-sampling mask. With local magnification on the red box, we observe that by learning the error correction module, the fine details, especially the low-contrast structures are better preserved, leading to a better reconstruction.

In Fig. 5, we also compare the MR images produced by the TLMRI, PANO, GBRWT and DC-CNN with their DECN counterparts on the 2D 20% under-sampling mask. The results are consistent with our observation in Cartesian under-sampling case.

We note the sparse and non-local prior based models are improved more significantly than the deep learning model DC-CNN, which can be attributed to the highly accurate reconstruction of DC-CNN, which leaves less structural residual information as demonstrated in Fig. 2.

We compare the DECN-based models with another two state-of-the-art deep learning compressed sensing MRI methods: Residual U-Net [19] and ADMM-Net [22] in Figs. 4 and 5. The Residual U-Net achieves the 34.39 dB in PSNR and 0.909 in SSIM compared to ADMM-Net with 28.12dB in PSNR and 0.727 in SSIM on 1D Cartesian 30% mask. On 2D Random 20% mask, the Residual U-Net achieves the 35.64 dB in PSNR and 0.878 in SSIM compared to ADMM-Net with 37.05dB in PSNR and 0.951 in SSIM. We observe the DC-CNN-DECN outperforms both methods.

**Fig. 6:**
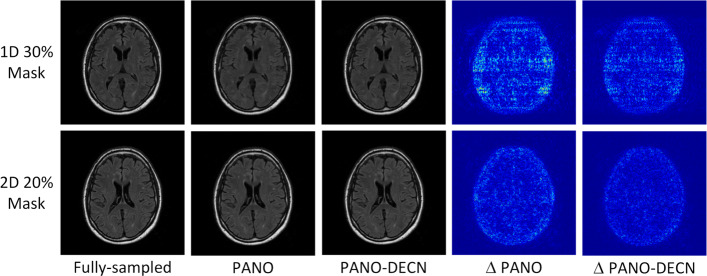
The experimental results on the MRBrainS13 datasets with PANO being guide module. The same 1D 30% and 2D 20% sampling masks are used in Figs. 4 and 5

We also test our DECN approach on the publicized MRBrainS13 dataset with PANO being guide module and show the visual results in Fig. 6. We observe the error correction strategy efficiently improve the reconstruction quality on this datasets. The objective results on PSNR and SSIM are shown in Table 3.

**Table 3:** The objective evalution on the regular CS-MRI inversions and their DECN frameworks

Measure	PSNR dB		SSIM	
Methods	PANO	PANO-DECN	PANO	PANO-DECN
1D 30%	39.24	41.49	0.953	0.9727
2D 20%	43.81	45.91	0.977	0.988

## Discussion

**Fig. 7:**
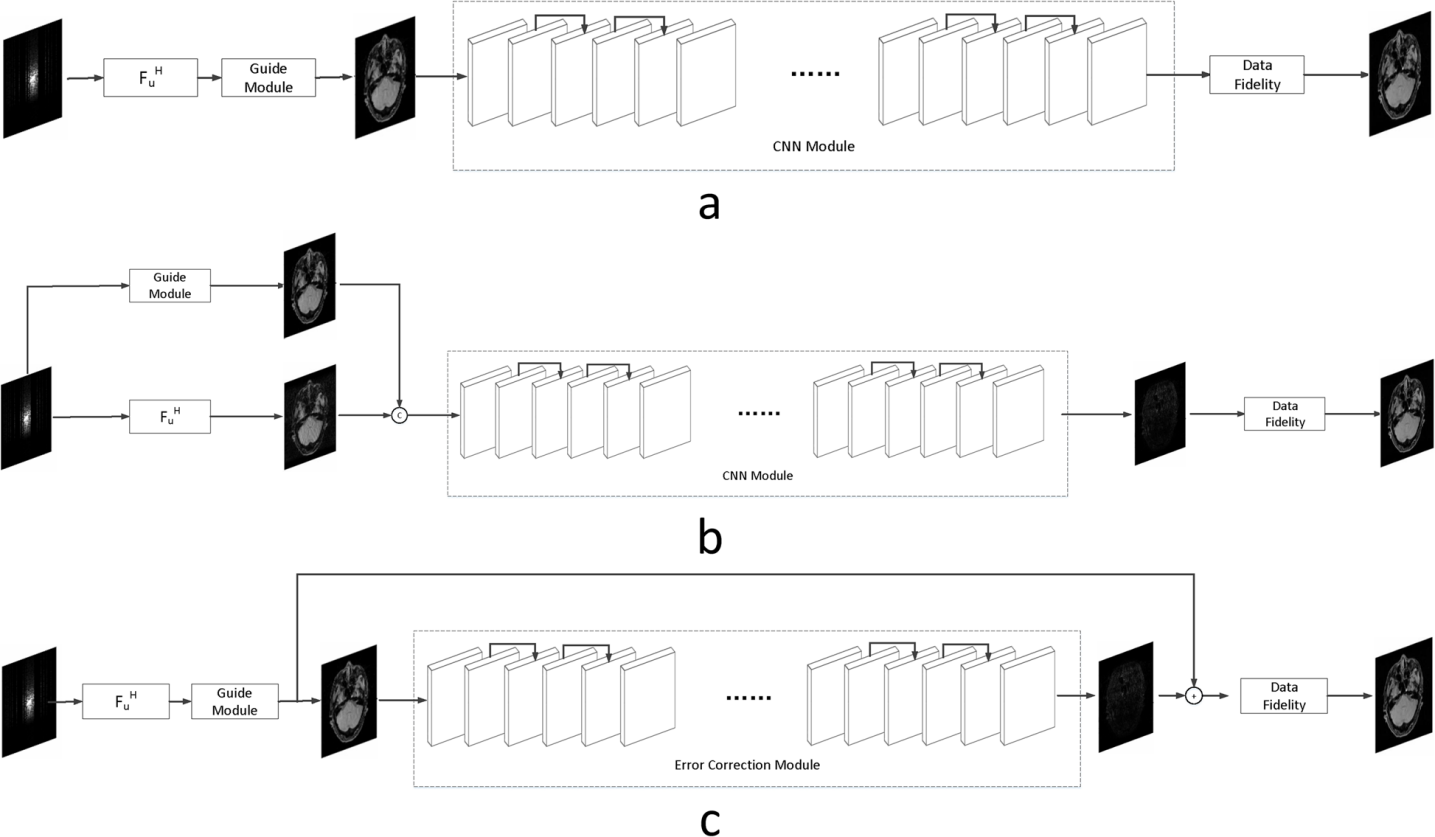
The compared baseline network architectures for the ablation study to evaluate the input concatenation and error correction strategies. **a** The DECN-NIC-NEC (DECN model without input concatenation and error correction), **b** The DECN-IC-NEC (DECN model with input concatenation and without error correction), **c** The DECN-NIC-EC (DECN model without input concatenation and with error correction)

To validate the architecture of the proposed DECN model, we conduct the ablation study by comparing the DECN framework with other Baseline network architectures in Fig. 7, which we refer the model in Fig. 7 as DECN-NIC-NEC (DECN with No Input Concatenation and Error Correction). With the guide module, a later cascaded CNN module learns the mapping from the pre-reconstructed MR image to the full-sampled MR image. Likewise, we name the models in Fig. 7 (DECN-IC-NEC) and Fig. 7 (DECN-NIC-EC). By comparing the DECN-NIC-NEC framework with the DECN-IC-NEC framework, we evaluate the benefit brought by the concatenating the zero-filled MR images and corresponding guide MR images as the input to compensate the information loss in the guide module. In Fig. 3, we give the illustration the information from zero-filled MR images and guide images can be shared. By comparing the DECN-NIC-NEC framework with the DECN-NIC-EC framework, we evaluate how the error correction strategy improves the reconstruction accuracy compared with simple cascade manner.

**Fig. 8:**
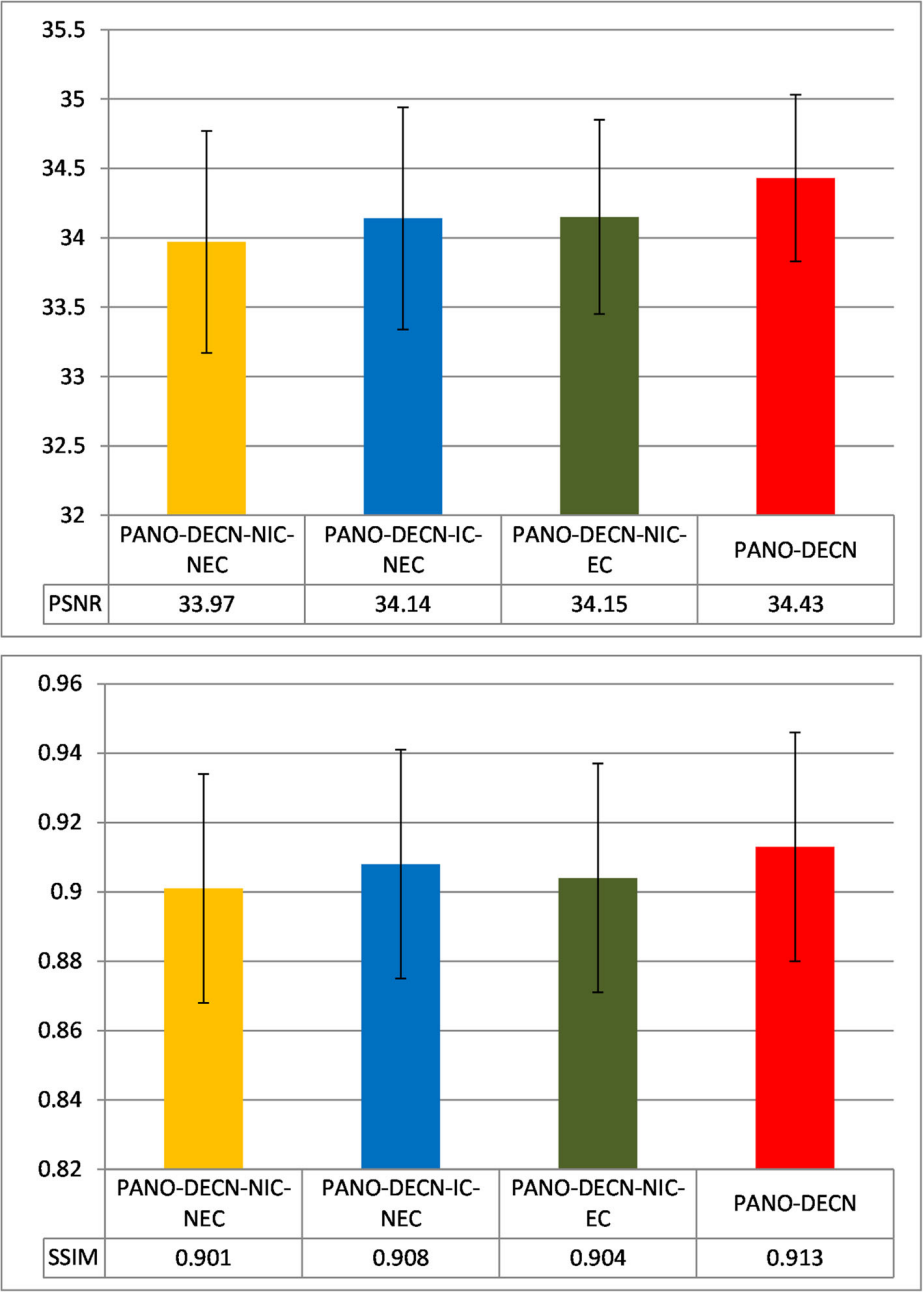
The PSNR and SSIM comparison of the baseline models and the proposed DECN model on the testing MRI datasets with standard deviation

Here we show the experimental results of the ablation study using PANO with the Cartesian under-sampling mask shown in Fig. 4 as the guide module. We give the averaged PSNR (peak signal-to-noise ratio) and SSIM (structural similarity index) results over the testing datasets in Fig. 8 and the standard deviation. We observe the PANO-DECN-IC-NEC and PANO-DECN-NIC-EC both outperforms the PANO-DECN-NIC-NEC with the similar margins about 0.2dB in PSNR. While the proposed PANO-DECN model with the input concatenation and error correction outperforms the PANO-DECN-NIC-NEC about 0.5 dB in PSNR. We can obtain the similar observations with other CS-MRI methods as guide module. The ablation study shows the input concatenation and error correction strategies can effectively improve the model performance in the DECN framework.

## Conclusions

We have proposed a deep error correction framework for the CS-MRI inversion problem. Using any off-the-shelf CS-MRI algorithm to construct a template, or “guide” for the final reconstruction, we use a deep neural network that learns how to correct for errors that typically appear in the chosen algorithm. Experimental results show that the proposed model achieves consistently improves a variety of CS-MRI inversion techniques.

## Data Availability

The MRBrainS13 dataset is publicly available.

## Abbreviations

BM3D: Block-Matching 3D MRI
CNN: Convolutional Neural Network
CS: Compressed Sensing
DC-CNN: Deep Cascade Convolutional Neural Network
DCT: Discrete Cosine Transform
DECN-NIC-NEC: DECN with No Input Concatenation and Error Correction
DECN: Deep Error Correction Network
DLMRI: Dictionary Learning Magnetic Resonance Imaging
FCSA: Fast Composite Splitting Algorithm
FDA: Food and Drug Administration
FLASH: Fast Low Angle SHot
GBRWT: Graph Based Redundant Wavelet Transform
k-t FOCUSS: k-t FOCal Underdetermined System Solver
MRI: Magnetic Resonance Imaging
NLR: Non-Local Regularization
PANO: Patch Based Non-Local Operator
PBDW/PBDWS: Patch Based Directional Wavelet
PSNR: Peak Signal-to-Noise Ratio
SSIM: Structural SIMilarity index RecPF: Reconstruction From Partial Fourier Data
ReLU: Rectified Linear Unit
TLMRI: Transform Learning Magnetic Resonance Imaging
TVCMRI: Total Variation *ℓ*_1_ Compressed MR Imaging
